# Effects of letrozole in combination with low-dose intramuscular injection of human menopausal gonadotropin on ovulation and pregnancy of 156 patients with polycystic ovary syndrome

**DOI:** 10.12669/pjms.326.11391

**Published:** 2016

**Authors:** Zhihua Chen, Mengzhen Zhang, Yuhuan Qiao, Junjuan Yang

**Affiliations:** 1 Zhihua Chen, Department of Gynecology, The First Affiliated Hospital of Zhengzhou University, Zhengzhou 450052, P. R. China; 2 Mengzhen Zhang, Department of Gynecology, The First Affiliated Hospital of Zhengzhou University, Zhengzhou 450052, P. R. China; 3 Yuhuan Qiao, Department of Gynecology, The First Affiliated Hospital of Zhengzhou University, Zhengzhou 450052, P. R. China; 4 Junjuan Yang, Department of Obstetrics and Gynecology, Women and Infants Hospital of Zhengzhou, Zhengzhou 450012, P. R. China

**Keywords:** letrozole, human menopausal gonadotropin, ovulation, pregnancy, polycystic ovary syndrome

## Abstract

**Objective:**

To explore the effects of letrozole (LE) in combination with low-dose intramuscular injection of human menopausal gonadotropin (HMG) on the ovulation induction and pregnancy of patients with polycystic ovary syndrome (PCOS).

**Methods:**

A total of 156 patients with PCOS infertility were randomly divided into an LE group, a clomiphene citrate (CC) group and an LE + HMG group (n= 52). LE and CC were orally taken according to the prescribed dosage on the 3rd-5th days of menstruation respectively, and 75 IU HMG was given through intramuscular injection. The ovulation induction parameters and pregnancy outcomes were observed.

**Results:**

The number of ovulation cycle of LE + HMG group was significantly higher than that of LE group (χ^2^=8.451, P<0.001). After injection of human chorionic gonadotropin, both endometrial thickness and number of mature follicles of LE + HMG group were significantly higher than those of other two groups (P<0.001), and the daily estradiol (E2) level was also higher (q=4.531, P<0.05). The pregnancy rate of LE + HMG group was 55.7%, which exceeded those of other two groups (compared to LE group, χ^2^=4.012, P<0.05). In LE + HMG group, the average medication cycle of clinically pregnant patients was (2.9 ± 0.3) weeks, which was significantly shorter than those of CC and LE groups (F=17.241, P<0.001).

**Conclusion:**

The regimen using LE in combination with low-dose intramuscular injection of HMG has satisfactory therapeutic effects on ovulation induction, short medication cycle and high clinical pregnancy rate, which is promising for treating patients with PCOS infertility.

## INTRODUCTION

Polycystic ovary syndrome (PCOS), as one of the most common endocrine disorders for women of childbearing age, has the incidence rates of 5%-10%, accounting for 30%-60% of anovulatory infertility.^1^ However, it remains difficult to design a proper regimen for the ovulation induction of PCOS patients. Clomiphene citrate (CC) is the first ovulation induction drug widely used in clinical practice, but it has a certain impact on the endometrium and cervical mucus. Besides, the pregnancy rate is relatively low despite high ovulation rate.^2^ In recent years, letrozole (LE), a drug originally used to treat breast cancer, is a third-generation aromatase inhibitor. LE can inhibit aromatase activity *in vivo* and reduce the conversion from androgen to estrogen, thereby partially releasing the negative feedback of estrogen to the hypothalamus and pituitary, and promoting the release of gonadotropin. As a result, follicular growth and ovulation are induced, and the conversion from androgen to estrogen is prevented.^3^

Human menopausal gonadotropin (HMG), which contains follicle stimulating hormone (FSH) and luteinizing hormone (LH), can secrete gonadotropin to promote follicle maturation, so as to stimulate ovulation and to accelerate the development of corpus luteum. Nevertheless, HMG can lead to multiple follicle development or cause multiple pregnancy and ovarian hyperstimulation syndrome (OHSS), so it is often used in combination with CC or LE in case of ineffective ovulation induction.^4^,^5^ Alternatively, high-dose HMG is used for ovarian hyperstimulation in combination with CC.^6^

In this study, 156 patients with PCOS infertility who were admitted in our hospital were selected and given different drug therapies, aiming to explore the effects of combined LE and HMG on the ovulation induction and pregnancy rate of PCOS patients.

## METHODS

### General information

All patients were admitted in our hospital between January 2013 and January 2015, who were not pregnant without contraception for over one year. The patients were aged between 23 and 38 years old, with a mean age of (26.9 ± 5.1). The durations of infertility were 1-12 years, (3.2 ± 1.0) years on average. All the cases were PCOS infertility patients in line with the PCOS diagnostic criteria of the 2003 Rotterdam Conference,^7^ i.e. at least two of the following three were met: 1) ovulation abnormality (sporadic ovulation or no ovulation) occurred after continuous monitoring for two or more natural cycles; 2) the results of B ultrasound showed polycystic ovary; 3) patients had hyperandrogenism or showed clinical manifestations of androgen excess, and those with androgen excess caused by other diseases such as adrenal hyperplasia, Cushing’s syndrome and androgen-secreting tumors were excluded. This study has been approved by the ethics committee of our hospital, and written consent has been obtained from all patients.

Through salpingography or hydrotubation under transvaginal B ultrasound and other examinations, all cases were confirmed to have tubal patency on at least one side. The semen of male was normal.

### Exclusion criteria

1) Infertility patients caused by non-PCOS ovulatory disorder or other factors; 2) patients with history of ovarian surgery or complication with endometriosis or pelvic adhesion; 3) patients complicated with liver, kidney or thyroid dysfunction; 4) patients who did not receive treatment after enrollment according to the established regimen or gave up in the midst of treatment.

The hormone (e.g. FSH, LH, estradiol (E2), testosterone (T), insulin and prolactin) levels in the venous blood of all patients were detected on the 2nd-4th days of menstruation. The patients with normal hormone levels were directly enrolled, and those with abnormalities were enrolled after neuroendocrine treatment. The patients were randomly divided into an LE group, a CC group and an LE + HMG group (n=52). They had similar age, duration of infertility, body mass index (BMI) and reproductive hormone levels (P>0.05) (Table-I).

**Table-I:** General information (x̄±s)

Group	n	Age (year)	Infertility duration (year)	BMI	Reproductive hormone level			

					FSH/IU·L^-1^	LH/IU·L^-1^	E2/pg·mL^-1^	T/mmol·L^-1^
LE group	52	26.4±4.2	3.4±1.1	22.4±4.5	7.1±1.5	5.7±1.1	57.7±13.5	1.41±0.5
CC group	52	27.1±4.7	3.2±0.7	23.4±1.5	6.9±1.2	5.5±0.9	54.5±12.7	1.45±0.3
LE + HMG group	52	27.7±5.2	3.3±1.3	22.6±2.6	6.8±1.1	5.9±1.2	61.5±15.8	1.42±0.2
F	-	0.703	0.311	0.515	0.211	0.163	1.635	1.785
P	-	>0.05	>0.05	>0.05	>0.05	>0.05	>0.05	>0.05

### Treatment methods

***LE group:*** The patients orally took 2.5-5.0 mg/d^-1^ LE (trade name: Fu Rui, Jiangsu Hengrui Medicine Co., Ltd.) on the 3rd-5th days of menstrual cycle for five consecutive days.

***CC group:*** The patients were orally administered with 50-100 mg/d^-1^ CC (trade name: Fertilan, Codal Synto Pharmaceutical Co., Ltd.) on the 3rd-5th days of menstrual cycle for five consecutive days.

***LE + HMG group:*** The patients orally took 2.5-5.0 mg/d^-1^ LE on the 3rd-5th days of menstrual cycle for five consecutive days. Starting from the day of oral administration of CC, 75 IU HMG (trade name: Lebaode, Livzon Group Livzon Pharmaceutical Co., Ltd.) was intramuscularly injected every other day for five consecutive days.

The three groups were expected to receive the treatment of ovulation induction for 4 to 6 cycles, and patients with poor outcomes were treated by assisted reproductive technologies.

### Treatment monitoring

Starting from the 10th day of menstruation, the growth conditions of follicles and endometrium in the patients were monitored once every other day by transvaginal B ultrasound, and then daily when the average diameter of the follicles was ≥16mm. When the average diameter of follicles was ≥18mm, the endometrial thickness, number of mature follicles and diameter of the largest follicle were recorded, and 5,000-10,000 U of human chorionic gonadotropin (HCG) (trade name: Fengyuan; Maanshan Fengyuan Pharmaceutical Co., Ltd.) was injected to induce ovulation. On the same day, the venous blood of patients was drawn to examine the LH, E2 and T levels, who were then guided to have sexual intercourse within 24 hour. B ultrasound examination was performed again 48 hour after HCG injection to observe follicle rupture and single follicle ovulation. If fetal heart beat was visible under transvaginal ultrasound on the 30th day after ovulation, the patients were diagnosed as clinical pregnancy.

### Observation indices

The numbers of completed cycle, ovulated cycle and single follicle-ovulated cycle in the three groups were recorded during ovulation induction, and the endometrium, follicle as well as reproductive hormone levels were observed on HCG injection day. In addition, whether OHSS occurred was recorded. After treatment, the clinical pregnancy (including normal pregnancy and abortion), multiple pregnancy and average medication cycle of clinically pregnant patients of the three groups were compared.

### Statistical analysis

All data were analyzed by SPSS17.0. The categorical data were expressed as mean ± standard deviation (±s). Three groups were compared by analysis of variance, and inter-group comparisons were performed by the SNK method. The numerical data were subjected to χ^2^ test for R×C table. When the theoretical frequency of at least one cell was lower than the expected value, the Fisher’s exact test was used. P<0.05 was considered statistically significant.

## RESULTS

### Success rates and follicle conditions of different regimens

The LE group had the most completed cycle, 52.4 of which had ovulation. The LE + HMG group completed the fewest cycles, with 65.3% of them ovulating. There were statistically significant differences (P<0.05). On HCG injection day, both the endometrial thickness and number of mature follicles of the LE + HMG group were significantly higher than those of the other two groups (P<0.001), but the follicle diameters were similar (P>0.05). All the three groups suffered from OHSS, but the incidences rates were similar (P>0.05). Of the 9 cases in the three groups, 7 were mild cases, manifested as abdominal bloating but without ascites, nausea or vomiting. Therefore, no special measures were taken. Five of the seven mild cases were completely relieved from OHSS in the 2nd-4th cycles, and the other two in the 5th cycle. One case of the LE + HMG group underwent severe OHSS, mainly manifested as hypoalbuminemia and electrolyte imbalance in the 3rd cycle. She was thereafter symptomatically treated until all the symptoms were eliminated (Table-II).

**Table-II:** Effects of different regimes on ovulation induction.

Group	n	Completed cycle	Ovulated cycle, %	Single follicle ovulation cycle	Monitoring on HCG injection day			

					Endometrial thickness (mm)	Number of mature follicles	Maximum follicle diameter (mm)	OHSS (case), %
LE group	52	215	113 (52.4)^a^	54	9.1±0.2	1.3±0.3	19.3±1.1	2 (3.8)
CC group	52	200	122 (61.0)	70	8.5±0.5	1.7±0.5	19.0±0.7	3 (5.7)
LE + HMG group	52	179	117 (65.3)^b^	77^b^	11.7 ± 1.2^a^	2.2 ± 0.2^a^	18.9±1.3	4 (7.7)
χ^2^/F	-	-	21.123	-	54.715	23.012	2.656	1.925
P	-	-	<0.001	-	<0.001	<0.001	>0.05	>0.05

a Comparisons between LE and CC groups, χ^2^=8.682, P<0.001;

b comparisons between the three groups, q=11.235, P<0.001.

### Effects of different regimens on hormone levels on HCG injection day

On HCG injection day, T level hardly changed, LH and E2 levels significantly increased, and there were no significant intergroup differences between LH levels. The LE + HMG group had significantly higher E2 level than those of LE and CC groups (P<0.05) (Table-III).

**Table-III:** Effects of different regimens on hormone levels on HCG injection day (x̄±s)

Group	n	LH/IU·L^-1^	E2/pg·mL^-1^	T/mmol·L^-1^
LE group	52	31.7±9.01	319.3±104.2	1.31±0.3
CC group	52	30.1±4.56	327.7±89.2	1.32±0.2
LE + HMG group	52	31.2±10.11	605.2 ± 112.1^a^	1.27±0.7
F	-	2.779	222.332	1.012
P	-	>0.05	<0.001	>0.05

a Comparisons between the three groups, q=4.235, P<0.05.

### Effects of different regimens on pregnancy

The pregnancy rate of the LE + HMG group was 55.7%, which was significantly higher than that of the CC group (P<0.05). There were no statistically significant differences in the abortion rate and multiple pregnancy rate among the three groups (P>0.05). The average medication cycle of the LE + HMG group was significantly shorter than those of the other two groups (P<0.05) (Table-IV).

**Table-IV:** Effects of different regimens on pregnancy.

Group	n	Clinical pregnancy (case), %	Abortion (case)	Multiple pregnancy (case)	Average treatment cycle
LE group	52	16 (30.8%)	2	2	4.1±0.5
CC group	52	17 (32.1%)	3	3	3.9±0.2
LE + HMG group	52	29 (55.7%)^a^	2	5	2.9 ± 0.3^b^
χ^2^/F	-	-	-	-	17.241
P	-	-	-	-	<0.001

a Comparisons between LE + HMG and LE groups, χ^2^=3.911, P<0.05;

b comparisons between the three groups, q=23.731, P<0.001.

## DISCUSSION

PCOS is a common reproductive endocrine disease among women, characterized by follicle developmental disorders, androgen excess and insulin resistance.^8^ For ovulation disorders caused by PCOS, CC and LE are used currently for ovulation induction. At present, CC remains the first-line drug for ovulation induction, with ovulation rates of 75% to 80%,^9^ but recent studies have shown that a considerable proportion of patients did not get desirable pregnancy outcomes after CC treatment.^10^,^11^ In the normal ovulation process, estrogen and progesterone play obvious regulatory roles, and CC has anti-estrogenic effects, but its exact mechanism has not been fully clarified yet.^12^ It has previously been reported that^13^,^14^ the chemical structure of CC is similar to that of estrogen, so it is capable of binding estrogen receptor (ER) and then competitively occupying the hypothalamic ER to inhibit the negative feedback of endogenous estrogen to the hypothalamus. In this study, the pregnancy rate of PCOS patients after treatment was 32.1%.

As the gene product of CYP19, aromatase can act on androstenedione generated from the adrenal cortex of adipose tissues to form estrone and testosterone in ovarian tissues to produce androstenedione, and then transform a part of androstenedione into estrone.^15^,^16^ Theoretically, LE may be superior to CC because it has no peripheral anti-estrogen effect. Al-Omari et al. found that^17^ the ovulation rate after using 2.5 mg LE reached 84.4% and the cycle pregnancy rate was 19%. Sun et al. reported that LE with a dose of 5.0 mg/d × 5 d had better follicle development and higher pregnancy rate than those of 2.5 mg/d × 5d LE, but increasing the dose to 7.5 mg/d did not effectively improve the treatment outcomes.^18^

The small impact of LE on the estrogen/progesterone ratio is conducive to maturation of the endometrium and receptivity increase.^19^ In this study, in the cycle with ovulation induction, the LE group had slightly fewer ovulation cycles and mature follicles than those of the CC group. The E2 hormone level of the CC group was slightly higher and the endometrium was thinner than those of the LE group. The OHSS incidence rate was also slightly higher than that of the LE group, but the clinical pregnancy rates of the two groups were similar. Moreover, the two groups had comparable completed cycles and cycles from treatment to clinical pregnancy.

Two regimens using HMG are used currently for ovulation induction: low-dose escalation and high-dose descending. The low-dose escalation regimen is most commonly used at present, which can reduce the incidence rates of OHSS and multiple follicles by timely adjusting the HMG dosage according to B ultrasound results.^20^ It is well-documented that^4^,^5^ single-use or high-dose HMG may lead to multiple pregnancy and OHSS, so it is recommended to minimize the dosage of HMG. We herein assessed the effects of LE in combination of small-dose injection of HMG on the ovulation induction of PCOS patients, and found the combination regimen had obvious advantages.

In summary, the regimen using LE in combination with low-dose injection of HMG every other day had a satisfactory effect on ovulation, short medication cycle and high clinical pregnancy rate, which provides a promising option for the treatment of patients with PCOS infertility. The regimen has many advantages, but ultrasound and related laboratory examinations are needed to monitor various indices during the treatment, thus increasing the cost of treatment. Furthermore, some patients have poor compliance in clinical practice. A large-sample-size and multi-center research is required to confirm the application value of the regimen in patients with ovulatory disorder infertility.
